# Xylanase Treatment Suppresses Light- and Heat-Induced Yellowing of Pulp

**DOI:** 10.1038/srep38374

**Published:** 2016-12-05

**Authors:** Daolei Zhang, Xuezhi Li, Meimei Wang, Yanxin Ye, Jian Du, Xianqin Lu, Jian Zhao

**Affiliations:** 1 State Key Laboratory of Microbial Technology, Shandong University, Ji-nan City, Shandong Province, 250100, P. R. China

## Abstract

Xylanase is commonly applied in pulp and paper industries to ease cost-related and environmental pressures. The effect of xylanase treatment on pulp bleaching is well-established, however, few studies were conducted on the effects of xylanase treatment in pulp yellowing, especially the mechanism of pulp yellowing inhibition by xylanase treatment. In this study, pure xylanase (EC 3.2.1.8) was applied to treat wheat straw chemical pulp (CP) and poplar chemi-thermo-mechanical pulp (CTMP) to determine their effects on pulp brightness and on light- and heat-induced yellowing. The xylanase treatment decreased the post-color number of the pulps during light- and heat-induced yellowing. However, differences were observed in the yellowing inhibition between the wheat straw CP and poplar CTMP. The changes in chemical components of pulps after the xylanase treatment, for example, lignin, hemicellulose, and HexA contents, and analysis of UV–vis absorption spectra and Fourier transform infrared-attenuated total reflectance spectrum were used to explore the pulp yellowing inhibition causes by the xylanase treatment.

The world’s total production of paper and paperboard reached approximately 400 million tons in 2012, and the global demand for paper and paperboard for packaging purposes has increased by 75% in the last five years^1^. As a high-level consumer of lignocellulosic resources, the papermaking industry should urgently expand its sources of raw material and improve its practical utilization of raw materials, especially in countries characterized by wood shortage, such as China^2^. Abundant non-wood materials with high annual yields per hectare and low cost of wheat straw and reed, for example, are raw materials well-suited to the pulp industry^2^,^3^,^4^,^5^. Straw is the most commonly utilized non-wood material in the pulp and papermaking industry. More than 70% of the total non-wood chemical pulp is bleached wheat straw pulp in China^6^. High-yield pulps (HYPs), such as chemi-thermo-mechanical pulp (CTMP), have been rapidly developed because of low cost and improved wood utilization^7^. Compared with chemical pulps, HYP fibers offer distinct papermaking advantages including bulk, opacity, stiffness, and printability^8^,^9^,^10^. In addition, HYP fibers can fully replace chemical pulps in certain high-value products.

The tendency to yellow, also called “brightness reversion,” is one major drawback to HYP and straw pulp application^11^,^12^. In general, pulp yellowing includes both light- and heat-induced yellowing. Pulps easily become yellow when exposed to daylight, indoor illumination, or high temperatures. For chemical pulps, heat-induced yellowing is the main form of brightness reversion. Lignin residues, carbonyl groups, xylan, hexenuronic acid (HexA) groups, and transition metal ions are involved in the yellowing reactions^13^,^14^. Residual lignin, the products of polysaccharide chain thermal decay and xylan are the main (although debatable) causes for heat-induced yellowing of chemical pulps^15^. Light-induced yellowing is principally associated with lignin-rich pulps (i.e., HYP). HYP discoloration is dominated by lignin-based reactions including the ketyl, phenoxyl, and phenacyl pathways, phenoxyl quinone redox cycle, and stilbene photodegradation^16^,^17^,^18^. Quinones and other chromogenic substances are produced through these pathways.

Many previous researchers have made valuable progress in methods of inhibiting pulp yellowing. UV absorbers have been proven to be particularly efficient. McGarry *et al*.^19^ discovered an inhibitor that helped maintain paper brightness for at least one year. Fluorescent whitening agents are another type of substance that can be applied to slow down pulp yellowing^20^, and studies have shown that chemical modification and paper coating are also effective methods of inhibiting yellowing^12^. Most of these methods are not appropriate in practice because of efficiency/cost ratios, technical difficulties, toxicity, and a few other reasons; thus, an environmentally friendly and low-cost process to decrease brightness reversion is necessary^12^.

Biotechnology applications (e.g., enzymatic improvement of pulp and enzyme-enhanced bleaching) are increasingly common in the pulp and papermaking industry. In addition, these applications show many notable advantages, including environmental friendliness. Ramos *et al*.^21^, for example, applied crude ligninolytic enzymes, which possess laccase, lignin peroxidase, and manganese peroxidase to sugar cane bagasse, to enhance pulping and paper properties. They also found that the brightness of enzyme-treated pulps increased by 2% compared with the control.

The xylanase-enhanced bleaching process has been particularly widely studied. This bleaching method is often used in industrial-scale production of pulps. In addition to improving the brightness of bleached pulps after bleaching, research has shown that xylanase treatment improves the brightness stability of bleached pulps^22^. Few studies have explored the mechanism of pulp yellowing inhibition by pure xylanase treatment despite assumptions of some studies regarding xylanase-enhanced bleaching mechanisms^23^,^24^. In these studies, however, crude xylanase or enzyme mixtures were often used. Thus, identifying the specific mechanism through xylanase treatment inhibiting pulp yellowing and improving pulp brightness stability is very difficult.

In this study, we investigated the effects of xylanase treatment on pulp light- and heat-induced yellowing and its inhibition yellowing mechanism. Pure xylanase (endo-1, 4-β-xylanase: EC 3.2.1.8) obtained by *purification of crude xylanase from liquid* fermentation of recombinant *Pichia* sp. GS115 was used in this study. Recombinant *Pichia* sp. GS115 harbors the xylanase gene from *Penicillium oxalicum* 114–2 to treat poplar CTMP and wheat straw CP.

## Results and Discussion

### Change in pulp chemical components by xylanase treatment

The chemical components of wheat straw CP and poplar CTMP before and after xylanase treatment at a xylanase dosage of 40 U/g pulp (oven dry weight) are listed in Table 1.

Table 1 shows that xylanase treatment reduced hemicellulose content in wheat straw CP compared with the control (*P* = 0.00, n = 3) because of depolymerization and dissolution of xylan in pulp. The decrease in hemicellulose content of wheat straw CP was higher than that of poplar CTMP after xylanase treatment under the same treatment conditions. This result is probably because wheat straw CP contained more hemicellulose compared with poplar CTMP and xylan is a primary hemicellulose sugar in wheat straw CP. The dissolution of xylan in pulp also benefitted the dissolution of low-molecular-weight lignin because of the destruction of lignin-carbohydrate compound (LCC) structure^25^, which results in lignin content reduction after xylanase treatment. Compared with the control, the sum of lignin, cellulose, and hemicellulose contents of treated wheat straw CP with xylanase was lower than 100%, for example, 91.6% for pulp sample and 95.7% for control, probably because xylanase treatment led to more oligosaccharides being produced in the pulp and easily extracted out in the water and ethanol extraction stage of chemical component analysis process. Some lignins were also easily extracted because the pulp structure is changed by enzyme treatment. This condition led to the decrease of analyzed contents of sugar and lignin. The HexA content of pulp also decreased after xylanase treatment by approximately 73.4% for wheat straw CP and 4.1% for poplar CTMP (*P* = 0.00, n = 3), as shown in Table 1.

After xylanase enzyme treatment, high-performance liquid chromatography (HPLC) analysis found that the filtrate from enzymatic stage mainly contained oligosaccharides and almost no monosaccharides. So the filtrate was first hydrolyzed with sulfuric acid to determine its sugar compositions and contents. Table 2 shows that xylose, arabinose, mannose, and acid-soluble lignin increased in the filtrate of wheat straw CP (*P* < 0.01, n = 3), and the contents of xylose and acid-soluble lignin also increased in the filtrate of poplar CTMP(*P* < 0.05, n = 3), although parts of monosaccharides and soluble lignin were inevitably lost because the reactions of monosaccharides degradation and condensation of lignin occurred during acid hydrolysis of the filtrates. The lignin condensation reaction would result in lignin with high molecular weight produced and precipitated out^26^. The results illustrated that a portion of the hemicellulose and lignin was dissolved during xylanase treatment. Higher contents of hemicellulose sugars in liquor treatment of wheat straw CP compared with that of poplar CTMP showed that the xylanase easily decomposed hemicellulose in wheat straw CP.

#### Effects of xylanase treatment on brightness of bleached pulp

**Figure 1:**
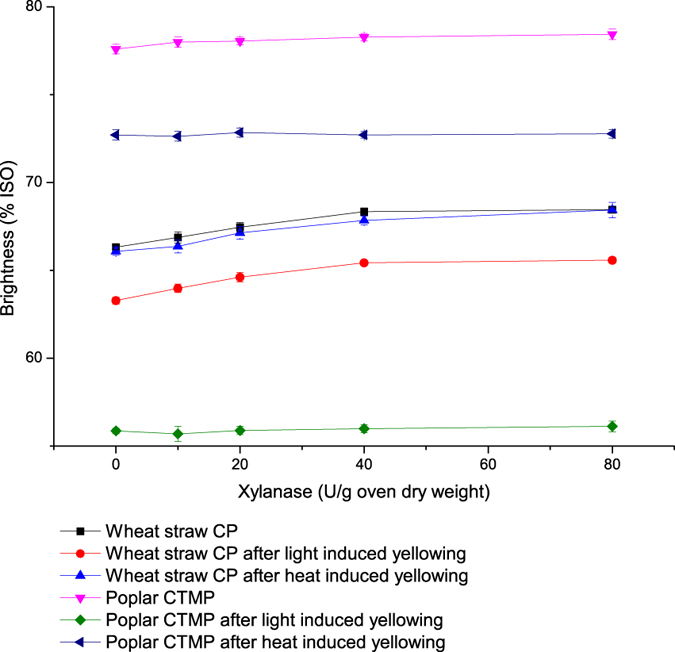
Effects of xylanase dosages on brightness of bleached pulps of wheat straw CP and poplar CTMP before and after heat- and light-induced yellowing. Data represent mean ± S.D., n = 3.

For wheat straw CP, xylanase treatment improved the brightness of the bleached pulp when the dosage reached 40 U/g of oven dry pulp as shown in Fig. 1. At this level, the brightness of the pulps increased by 3.0% (un-aged), 3.4% (light-induced yellowing), and 2.7% (heat-induced yellowing) (*P* < 0.01, n = 3) compared with the control. The change in brightness of bleached poplar CTMP was obviously less than that of bleached wheat straw CP. For un-aged and light-aged poplar CTMP samples, the bleached pulp brightness at a xylanase dosage of 40 U/g increased by 0.9% and 0.2% compared with the control (*P* < 0.01, n = 3), respectively. For heat aging of bleached poplar CTMP, xylanase treatment exhibited no effect on the pulp brightness.

Light- and heat-treatment caused the pulps to become yellow, and the brightness of the pulps after light aging significantly decreased under the experimental conditions, especially for poplar CTMP (Fig. 1), probably due to the disparity of contents and properties of lignin in the bleached pulps.

#### Effects of xylanase treatment on brightness stability

**Figure 2:**
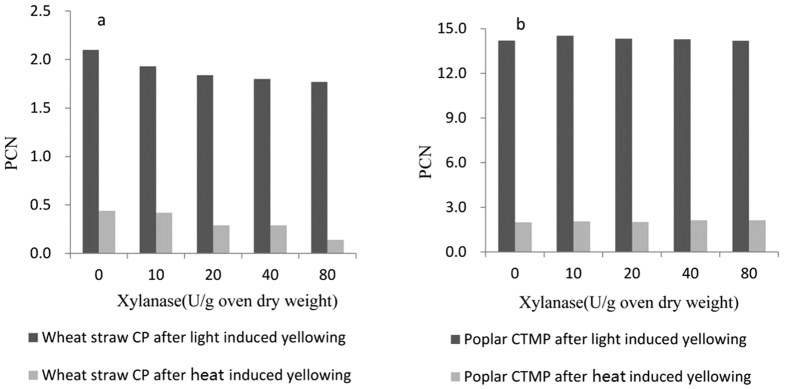
Effect of xylanase dosage on PCN of bleached pulps of wheat straw CP (**a**) and poplar CTMP (**b**) during heat- and light-induced yellowing.

Post color number (PCN) is an index that expresses the degree of brightness reversion of pulps before and after aging. Figure 2(a) shows that, along with the increase of xylanase dosage, the PCNs of wheat straw CP clearly decreased after light- and heat-induced yellowing. The PCNs of the bleached pulp samples at 40 U/g of xylanase dosage after light and heat aging decreased by 14.3% and 34.1% respectively compared with the control (the control corresponds to a dosage of 0 U/g). However, xylanase treatment exhibited little effect on the PCN of poplar CTMP after light and heat aging compared with wheat straw CP (Fig. 2b). A likely cause of the phenomenon is attributed to more hemicellulose and HexA contents in wheat straw CP and more lignin in poplar CTMP. Hemicellulose polysaccharides, because of their relatively low degree of polymerization, were easily hydrolyzed to low-molecular carbohydrate compounds, which subsequently underwent dehydration and condensation reactions to form chromogenic substances^27^,^28^; HexA is also easily degraded during heat- and light-induced yellowing to generate colored compounds^27^. Increased xylanase dosages led to enhanced hemicellulose removal, for example, hemicellulose content of wheat straw CP is 13.6% and 12.8% when xylanase dosages are 20 and 80 U/g, respectively, thus decreases pulp PCN. Poplar CTMP was more easily yellowed under irradiation condition compared with wheat straw CP (Fig. 2) because light-induced yellowing are mainly related to lignin-based reactions^29^, and the high lignin content in poplar CTMP masked the yellowing inhibition effect that caused by the decreased hemicellulose after xylanase treatment.

#### Yellowing inhibition mechanism by xylanase treatment

##### UV–vis DR Spectra of pulps

UV–vis DR spectral analysis is a quick and simple method to examine brightness stability. The reflectance spectra are sensitive to changes in chromophores and colorless UV-active structures induced by aging^12^,^14^. When the reflectance spectra were converted to absorbance (K/S) spectra via the Kubelka–Munk equation, information regarding chemical changes in the pulps that caused yellowing can be directly obtained^30^.

**Figure 3:**
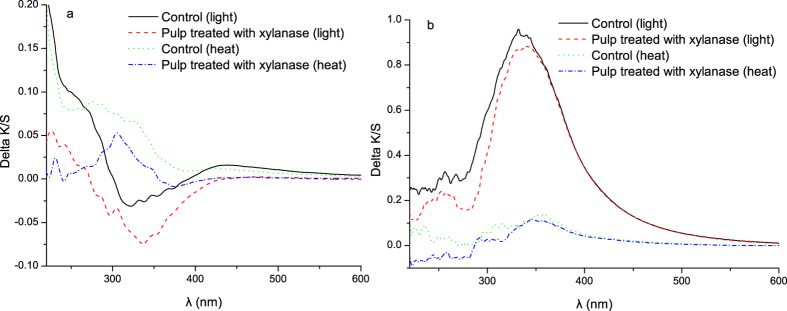
Difference UV-Vis absorption (ΔK/S) spectra of wheat straw CP (**a**) and poplar CTMP (**b**) before and after heat- and light-induced yellowing (xylanase dosage: 40 U/g).

The UV–vis DR spectra of untreated and treated unbleached wheat straw CP with xylanase before and after heat- and light-induced yellowing are presented in Fig. 3(a).

For light-induced yellowing wheat straw CP (Fig. 3a), ΔK/S of the pulp treated with xylanase was lower than that of untreated pulp. This result indicates that xylanase treatment decreased the content of chromophores and its precursors. In addition, signal intensity at 300 nm to 350 nm was lower than that at other wavelengths. This change may have been caused by the dissolution of lignin (coniferaldehyde moiety), or degradation of carbonyl groups in lignin, which had been proved by literatures^30^,^31^. For heat-induced yellowing of wheat straw CP (Fig. 3a), the ΔK/S of the pulp treated with the xylanase was also lower than that of the untreated pulp. This result implies that less chromophores were generated after heat aging by xylanase treatment. In particular, the ΔK/S of the pulp at approximately 285 nm after heat aging significantly decreased compared with untreated sample. This change may have been caused by the degradation of HexA^32^ (Table 1). The HexA is converted to UV-active compound (5-formyl-2-furancarboxylic acid: FFA), and this compound (FFA) can then produce chromophores during heat aging^32^.

Compared with the controls, signal intensity of xylanase-treated samples at approximately 285 nm was much lower regardless of light or heat aging. This result might be caused by the decrease of guaiacyl aromatic ring of lignin^33^. For poplar CTMP (Fig. 3b), a noticeable absorption was observed at 340 nm for xylanase-treated pulp and the untreated pulp after light-induced yellowing. This condition indicates that irradiation exhibited sizeable influence on the brightness reversion for lignin-rich pulps. For heat-aged poplar CTMP, the slightly lower ΔK/S for xylanase-treated samples compared with that of the untreated sample showed that xylanase treatment slightly decreased chromophore generation during heat aging. However, the inhibition effect on heat-induced yellowing of the pulp was very weak. This result was consistent with PCN changes as shown in Fig. 2b. As shown in Fig. 3b, the signal intensity of control below 350 nm was higher than that of xylanase-treated pulp. However, no difference was observed between the untreated and treated samples in the visible region in both light- and heat-induced yellowing. This condition shows that xylanase treatment of poplar CTMP decreased the content of the UV-active compound. On the contrary, the formation of chromophores was not inhibited.

##### Fourier transform infrared-attenuated total reflectance (FTIR-ATR) spectrum analysis

**Figure 4:**
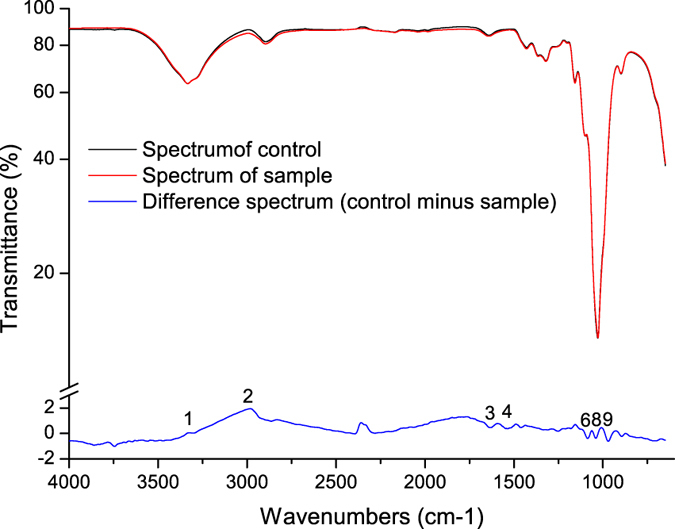
FTIR-ATR and difference spectra of wheat straw CP untreated pulp (control) and treated pulp with xylanase.

**Figure 5:**
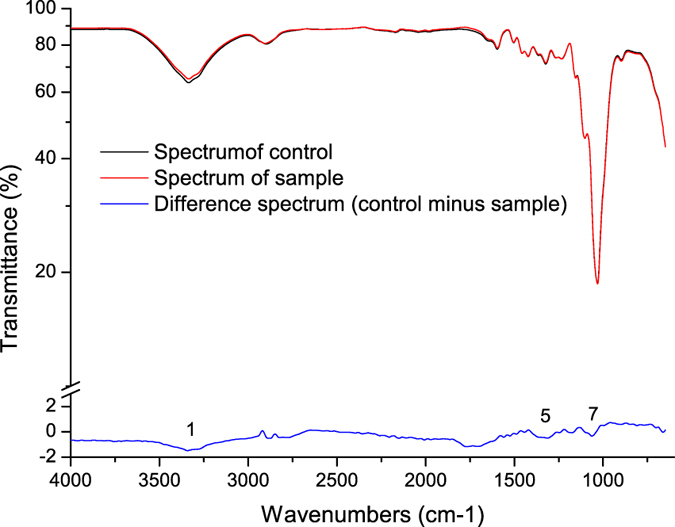
FTIR-ATR and difference spectra of poplar CTMP untreated pulp (control) and treated pulp with xylanase.

FTIR-ATR and their difference spectra of unbleached wheat straw CP and unbleached poplar CTMP before and after xylanase treatment are shown in Figs 4 and 5. The difference spectra (Fig. 4) at approximately 3333 and 2899 cm^−1^ region (peaks 1 and 2) correspond to O–H and C-H vibrations. The signal intensity at 1638 cm^−1^ (peak 3) relating to the conjugated carbonyl groups decreased. This condition is consistent with the analysis results for ΔK/S, i.e., xylanase treatment decreased the content of lignin as shown in Fig. 3a. Thus, the brightness stability of wheat straw CP was enhanced. Change of signal at 1509 cm^−1^ (peak 4) corresponding to aromatic skeletal vibrations indicated that the lignin content in wheat straw CP decreased after xylanase treatment. The difference spectra at 1104 cm^−1^ (peak 6) corresponding to C–O vibrations were likely related to the decreases in hemicellulose content after xylanase treatment. This observation was further confirmed by a decreased intensity at 897 cm^−1^ (peak 9). This result corresponds to anomeric carbon (C1) vibrations in hemicellulose. The decrease in absorption at 1031 cm^−1^ (peak 8) corresponding to C–H (guaiacyl) stretching vibrations indicated that the lignin in wheat straw CP decreased after xylanase treatment. This result is consistent with the results of UV–vis DR spectral analysis at approximately 280 nm.

The difference spectra of poplar CTMP were smoother than that of wheat straw CP, as shown in Fig. 5. This result indicates that xylanase treatment exhibits little influence on poplar CTMP. The adsorption at 1052 cm^−1^ (peak 7) relating to C–O stretching vibrations and 1315 cm^−1^ (peak 5) to CH_2_ vibrations in cellulose and hemicellulose decreased, suggesting that the hemicellulose was partly hydrolyzed during xylanase treatment. Thus, pulp brightness stability was improved.

## Conclusions

Pure xylanase treatment prior to H_2_O_2_ bleaching of wheat straw CP increased the pulp brightness and inhibited heat- and light-induced yellowing of pulp. However, the treatment exhibited only a slightly positive influence on pulp brightness and brightness stability of poplar CTMP. Changes in hemicellulose, HexA, and lignin contents in xylanase-treated pulps, as well as differing lignin structures and active groups related to chromophores and precursors, were the causal factors in xylanase treatment’s ability to inhibit heat- and light-induced yellowing of pulp while improving pulp brightness.

## Materials and Methods

### Materials

#### Pulps

Poplar CTMP was kindly provided by Huatai Paper Co., Ltd. (Shandong, China). Unbleached neural sulfite wheat straw pulp with pH value of 7 to 8 was provided by Shan Dong Tranlin Group (Shandong, China).

## Methods

### Xylanase production

Xylanase PDE_08094, consisting of 388 amino acid residues with a calculated mass of 41.41 kDa, was produced by liquid fermentation of *Pichia* GS115 from *P. oxalicum* 114–2. The xylanase enzyme belongs to the GH10 family, which means that this enzyme contains carbohydrate-binding modules (CBMs). CBMs of the enzyme can anchor biocatalysts to the plant cell wall and potentiate catalytic effect^34^. *Pichia* sp. GS115 were grown in yeast extract peptone dextrose medium (1% yeast extract, 2% peptone, and 1% dextrose) for 24 h at 30 °C, 200 rpm to inoculate the shake flask. Up to 1% (v/v) bacterial suspension was added to the 50 mL shake flask containing 5 mL buffered minimal glycerol-complex medium (BMGY, 1% yeast extract, 2% peptone, 1.34% yeast nitrogen base, 0.4 μg/mL biotin and 1% glycerol, 100 mM potassium phosphate, pH 6.0) and cultured for 18 h at 30 °C, 200 rpm. Cells were collected by centrifugation at 3500 × *g* for 5 min at 4 °C, re-suspended in 50 mL buffered methanol-complex medium (BMMY, using 0.5% methanol instead of 1% glycerol in BMGY) (OD_600_ = 1.00), and cultured under the same conditions. Methanol was added every 24 h (1% v/v of final concentration) to maintain induction. After 72 h of culture with methanol, the crude enzyme was obtained by centrifugation (3500 × *g*, 5 min, 4 °C). Carboxymethyl cellulase (CMCase) and filter paper activities were not detected in the enzyme solution. An AKTA Purifier 100 (GE, Sweden) was used to purify the crude enzyme with the HiPrep 26/60 Sephacryl S-100HR column (GE Healthcare, Sweden) to remove contaminating proteins and other impurities. Fractions were collected and concentrated with ultrafiltration equipment (Millipore Pellicon XL, USA) with a Biomax 10 membrane (polyethersulfone) and stored at 4 °C. The purified enzyme migrated as a single band on sodium dodecyl sulfate-polyacrylamide gel electrophoresis.

### Pulp Treatment with Xylanase

Approximately 1 g pulp (oven dry weight) was washed with 20 mL tap water twice, then, washed with 20 mL sodium acetate buffer (50 mM, pH 4.8) in respective nylon filter bags (250 μm) and transferred into polyethylene bags. Pulp concentration was adjusted to 10% with sodium acetate buffer. The xylanase dosages used were 10, 20, 40, and 80 U/g pulp (oven dry weight). Xylanase treatment was conducted in a thermo-stated water bath at 50 °C for 2 h. During this time, the polyethylene bags were kneaded once every 20 min to ensure a uniform reaction. After incubation, the pulps were filtered through a Buchner funnel, and the filtrates were analyzed for lignin and sugar contents. The solid residues (pulps) were washed with 20 mL tap water three times to remove residual enzymes. Control samples were treated with the same conditions except for adding xylanase.

### Pulp Bleaching

Xylanase treated and the control pulps’ concentration were adjusted to 10% and treated with 0.5% (w/v) EDTA at 80 °C (water bath) for 2 h in polyethylene bags. After metal ion chelation, the pulp suspensions were conditioned with 1.5% (w/v) NaOH, 3% (w/v) Na_2_SiO_3_, 0.5% (w/v) MgSO_4_, and 3% (w/v) H_2_O_2_. Consistency was adjusted to 10%, and pH to 10 to 11. Treatment was carried out at 80 °C for 2 h.

### Pulp Yellowing

Light-induced yellowing was examined by placing samples in an aging box with four UVB-340 lamps (Xue Laite, China) that emit light in UV–vis range (λ: 295–350 nm) with λmax = 340 nm. A cooling fan was used to minimize the heating effect of the light source. Temperatures detected in the aging box were approximately 27 °C. Pulp samples were located in points that had same light intensity (4.6 ± 0.1 μW/cm^2^, determined by ultraviolet irradiation) and aged for 24 h. The pulps were aged in a dry air circulating oven at 105 °C for 24 h to examine heat-induced yellowing. After yellowing, all treated samples were loaded in opaque bags and balanced for 4 h before pulp brightness analysis. At least three parallel test sheets were used both for light- and heat-induced yellowing.

### UV Absorption and FTIR-ATR Spectra

UV absorption of the filtrates after enzyme treatment and the control were measured with a Shimadzu UV-2550 spectrometer (Shimadzu, Japan). FTIR-ATR spectra of pulps were recorded with a Tensor 27 spectrophotometer (Bruker, Germany) under the following conditions: ZnSe crystals, 45° angle of incidence, 2 cm resolution, 32 independent scans, and a scan range of 4000–650 cm^−1^. The infrared difference spectra were obtained by transmittance spectrum of pulp samples without xylanase treatment (control) minus that of sample treated with xylanase.

### Ultraviolet–visible Diffuse Reflectance (UV–vis DR) Spectra

UV–vis DR spectra of pulp samples were recorded in a UV-2550 spectrophotometer equipped with an ISR-2200 integrating sphere, with BaSO_4_ as a background reference. The spectral resolution was set to 5 nm, and the data were pitched every 2 nm. The reflectance spectra were converted to absorbance spectra (K/S) using the Kubelka–Munk equation^31^, in which, the K and S are the absorption and scattering coefficients. The change of scattering coefficient (S) is very small when the handsheets are thicker than 30 g/m^2^. Thus, the scattering coefficient (S) in the Kubelka–Munk equation is constant^30^. In this case, K/S is directly related to the concentration of the chromophores and the UV-active substances within the handsheet^12^.

The absorption difference spectra (ΔK/S vs. wavelength) were obtained by subtracting the absorbance spectra (K/S) of an un-aged sample from that of the same sheet aged for 24 h. The difference in ΔK/S represents a net change in brightness reversion (according to chromophores and UV-active substances). ΔK/S, which is approximately linear to changes in chromophores or UV-active substances can be assumed according to Kubelka–Munk theory.

## Analytical Methods

### Enzyme Assays

Xylanase activity was assayed in a colorimetric tube of 25 mL consisting of 1.5 mL sodium acetate buffer (50 mM, pH 4.8) containing 1% (w/v) of oat spelt xylan and 0.5 mL of appropriately diluted enzyme. The colorimetric tubes were incubated at 50 °C for 30 min. After incubation, 3 mL 3,5-dinitrosalicylic acid was added and boiled for 10 min, then cooled to room temperature in ice bath, and added with distilled water to 25 mL. The absorbance at 540 nm was determined by using the Shimadzu UV-2550 spectrometer. Controls were treated with the same conditions except for adding 0.5 mL sodium acetate buffer instead of enzyme. Sugar concentration was interpolated from standard curve of xylose. One unit of enzyme activity was defined as the amount of enzyme that released 1 μ mol sugar per minute under the assay conditions^35^.

### Pulp Brightness and Post Color Number

ISO brightness of pulp samples^36^ was measured using a brightness color tester (YQ-Z-48A, Hangzhou Light Instrument Development Co., Ltd.). All reported brightness values represent an average of three separate measurements. The degree of brightness reversion is expressed by PCN, which is calculated as follows:


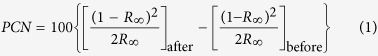


where *R*∞ denotes the ISO brightness of a thick pad. PCNs reported in this study were calculated using the mean brightness values of parallel samples.

### Chemical Composition Analysis

Chemical compositions of pulps, such as cellulose, hemicellulose, acid-soluble lignin (ASL) and acid-insoluble lignin, and extractives, were determined according to National Renewable Energy Laboratory (NREL, USA) analytical methods^37^. The filtered acid-hydrolysis liquid pH values were adjusted to 5–9 with powder Ba (OH)_2_, centrifuged for 15 min. The contents of different sugars in the supernatant were measured by HPLC (Shimadzu, Kyoto, Japan) with a refractive index detector (Shimadzu) in an Aminex HPX-87P column (Bio-Rad, Hercules, CA, USA) run at a flow rate of 0.5 mL/min at 78 °C (detector temperature: 40 °C), with filtered (0.22 μm) and degassed ultra-pure water as mobile phase. The samples were passed through a 0.22 μm polyether sulfone filter with 1 mL injector before HPLC analysis.

Cellulose and hemicellulose contents were calculated using Eqs. (2) and (3):


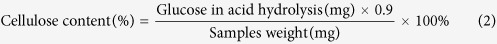



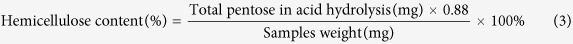


ASL was measured by a UV-2550 spectrophotometer (Shimadzu) after appropriate dilution of the filtrate at 240 nm; then, the relative absorption was calculated by multiplying the absorbance with the dilution factor^37^. ASL and total lignin content were calculated using Eqs (4), (5), and (6):


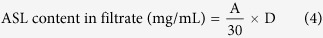



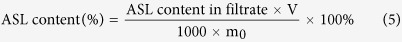



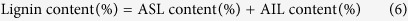


where *A* represents the filtrate absorbance, 30 is the absorption coefficient of wheat straw and poplar materials (L/[g·cm]), *D* is the filtrate dilution multiple, *V* is the hydrolysate volume (mL), and *m*_0_ is the oven dry weight of pulp (g).

Filtrates from enzymatic treatment step were centrifuged for 15 min, boiled for 10 min to deactivate enzyme, hydrolyzed with sulfuric acid (4%, w/v) for 1 h at 121 °C in autoclave, and passed through a 0.22 μm filter. Then, hydrolysates and acid-soluble lignin in filtrate were measured by HPLC and UV spectroscopy analysis at above identical conditions.

HexA contents of the pulps were measured by UV spectroscopy using the same method of Chai *et al*.^38^. Test sheet (approximately 50 μg) with known moisture content was accurately weighed and hydrolyzed in 10 mL hydrolysis solution (22 mM mercuric chloride and 50 mM sodium acetate), good shaking by hand, and incubated at 60 °C for 30 min. Then, UV absorptions at 260 and 290 nm were detected. The same hydrolysis solution without sample was used as the blank in absorption measurements. The HexA content can be calculated as Eq. (7):


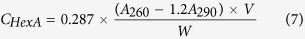


where 0.287 is the calibration factor (μmol·cm/L); A_260_ and A_290_ are the absorbance at 260 and 290 nm [L/(g·cm)]; *V* represents the volume of solution (mL); and *w* is the weight of sample detected (g). All the experiments of chemical composition analysis were carried out independently in triplicate. The results presented were mean of the three values.

### Statistical analysis

A t-Student one-tail test was performed with the software Microsoft Office 2010 Excel (Microsoft, USA). The mean values, standard deviations, and *P* values were calculated in all quantitative analysis.

## Additional Information

**How to cite this article**: Zhang, D. *et al*. Xylanase Treatment Suppresses Light- and Heat-Induced Yellowing of Pulp. *Sci. Rep.* **6**, 38374; doi: 10.1038/srep38374 (2016).

**Publisher's note:** Springer Nature remains neutral with regard to jurisdictional claims in published maps and institutional affiliations.

## Figures and Tables

**Table 1:** Chemical components of wheat straw CP and poplar CTMP before and after xylanase treatment.

Pulps		Lignin (%)	Cellulose (%)	Hemicellulose (%)	HexA (μmol/g)
Wheat straw CP	Control	11.5 ± 0.4	68.2 ± 0.5	16.0 ± 0.2	6.4 ± 0.3
	Xylanase	9.9 ± 0.3	68.7 ± 0.4	13.0 ± 0.2	1.7 ± 0.2
Poplar CTMP	Control	34.1 ± 0.3	44.5 ± 0.3	14.3 ± 0.1	4.9 ± 0.3
	Xylanase	34.9 ± 0.3	44.3 ± 0.2	14.1 ± 0.2	4.7 ± 0.3

Data represent mean ± S.D., n = 3.

**Table 2:** Chemical components changes of supernatants from xylanase treatment stage after xylanase treatment (mg/mL).

Pulps	Xylose	Arabinose	Mannose	Soluble Lignin (×10^3^)
Wheat straw CP	1.909 ± 0.003	0.135 ± 0.002	0.118 ± 0.003	93.33 ± 1.90
Poplar CTMP	1.332 ± 0.002	0.000	0.000	0.20 ± 0.03

The samples was treated with xylanase of 40 U/g. Data represent mean ± S.D., n = 3.
